# Parsonage-Turner syndrome, affecting suprascapular nerve and especially to infraspinatus muscles after COVID-19 vaccination in a professional wrestler, a case report and literature review of causes and treatments

**DOI:** 10.1186/s12883-024-03694-0

**Published:** 2024-06-05

**Authors:** Soheila Ganjeh, Hamidreza Aslani, Khosro Khademi Kalantari, Mohammad Mohsen Roostayi

**Affiliations:** 1 Student Research Committee, Department of Physical Therapy, Faculty of Rehabilitation, Shahid Beheshti University of Medical Sciences, Tehran, Iran; 2 Department of Orthopedics, Knee and Sport Medicine Education and Research Center, School of Medicine, Shahid Beheshti University of Medical Sciences, Tehran, Iran; 3 School of Rehabilitation, Shahid Beheshti University of Medical Sciences, Tehran, Iran

**Keywords:** Parsonage-Turner syndrome, Brachial plexus neuritis, Suprascapular nerve lesion, COVID-19

## Abstract

**Background:**

Acute peripheral neuropathy, also known as Parsonage-Turner syndrome or neuralgic amyotrophy, mostly affects the upper brachial plexus trunks, which include the shoulder girdle. It is typically accompanied by abrupt, intense pain, weakness, and sensory disruption. The etiology and causes of this disease are still unknown because of its low prevalence, however viral reactions-induced inflammation is one of its frequent causes.

**Case presentation:**

Here, we introduce a professional wrestler patient who was diagnosed with PTS after vaccination and was treated, and we review some articles in this field.

**Conclusion:**

When it comes to shoulder-girdle complaints and pain, Parsonage-Turner syndrome can be a differential diagnosis. Corticosteroids during the acute period, followed by physical therapy, appear to be an efficient way to manage pain, inflammation, muscular atrophy, and the process of recovering to full nerve regeneration.

## Introduction

As a differential diagnosis, nerve injuries are frequently disregarded as a possible cause of unusual shoulder discomfort and can be challenging to diagnose clinically, which can leave the patient with a protracted impairment [1]. Lesions of the anterior horn of the spinal cord, nerve root [2], plexus such as acute brachial plexus neuritis (BN) [3], peripheral nerve lesion such as Quadrilateral space syndrome [4, 5] and suprascapular entrapment [6] can all lead to these injuries. The causes of entrapment can include arteriovenous malformation such as those found in the spinoglenoid notch that impinge on the suprascapular nerve and cause atrophy of the infraspinatus muscle., or some cyst that compress the nerves [7], thickened or ossified ligaments [8] and massive rotator cuff tears [9].

Because there is a wide range of illnesses that can cause these injuries, a correct diagnosis may be challenging. Julius Dreschfeld originally described Parsonage-Turner syndrome (PTS) [10], an uncommon upper extremity condition that is often referred to as idiopathic brachial plexopathy or neuralgic amyotrophy (NA) [11]. The first set of "localized shoulder girdle neuritis" cases was reported by Spillane in 1943 [12], but in 1948, M.J. Parsonage and John W. Alden Turner introduced the condition with specifics regarding its clinical features [13]. Extreme neuropathic pain episodes, fast multifocal weakening, and upper limb atrophy are the hallmarks of PTS, a unique peripheral nervous system illness [13, 14]. Two different kinds of neuralgic amyotrophy—idiopathic and hereditary—exist, with an immune-mediated mechanism [15, 16]. Autosomal-dominant recurrent neuropathy affecting the brachial plexus due to abnormalities in a septin family gene characterizes the hereditary form of the illness. The hereditary form of the illness has been identified in multiple families with mutations in the gene septin 9 on chromosome 17q23 [16].

Its prevalence is 1.64 per 100,000 subjects [17], while two to three subjects per 100,000 individuals [18] and one per 1000 people were reported in some earlier investigations [19]. Taking into account possible misdiagnoses, the actual annual incidence seems to be at least 20–30 cases per 100,000 subjects [20]. Men are more likely than women to experience it [21] and the second or third decade of life is when it occurs most frequently [14]. It may be bilateral in 30% of cases [22]. Regretfully, data indicates that, in three out of four cases, this ailment is identified within 28 weeks of the sickness beginning [19]. Health care providers, particularly those who see patients directly, are therefore in a crucial position to detect this illness as soon as feasible.

A six-month-old infant was described as the youngest patient with PTS. One week following a viral illness, the infant experienced right upper extremity weakness, primarily in abduction and elevation, with the C5–C7 nerve roots showing the most involvement. Following a primary and neurologic examination, the brachial plexus showed no aberrant signals or discernible mass effect. With a diagnosis of BN, the infant was given prednisolone treatment and directed to an occupational therapist. Over the course of ten months, the youngster received an intensive therapy regimen, and her right upper extremity function significantly improved [23].

According to Parsonage and Turner, the pathological process in many PTS cases was in one or more peripheral nerves, and they proposed that the syndrome be referred to as NA until its etiology and pathophysiology are known [13, 24]. Put another way, PTS usually affects upper brachial plexus trunk with or without long thoracic nerve involevement [13, 22, 25] and middle brachial plexus trunks [26], not the entire plexus [24]. While PTS sequel can impact any nerve or nerves within the brachial plexus, data indicate that the major nerve affected in these cases is the suprascapular, axillary, musculocutaneous, long thoracic, and radial nerves. [27, 28]. Up to 78% of subjects with PTS also have involvement of the sensory nerves in addition to motor nerve complaints, with paresthesia and hypoesthesia being the most typical symptoms [22]. Persistent, severe, mostly unilateral shoulder girdle pain that originates at the top of the shoulder blade and may extend down the outside side of the upper arm or into the neck is the typical definition of PTS [13, 26]. Pain, may last for two hours to eight weeks, followed by sporadic paresis of the upper limb and shoulder girdle [22, 26, 29].

It can be diagnosed by clinical sign and symptoms [30], MRI, EMG and nerve conduction testing [7]. As previously stated, the etiology of it is unknown [13, 31], however some theories put forth in the literature include hereditary [16, 32, 33], infection that is most common than other [31, 34–39], vaccination [17, 40–44], surgery [45], autoimmune [15, 46], peri-partum, peri-operative [11], trauma and vigorous physical activity [44].

The prognosis is generally better for patients with upper trunk involvement than for those with lower trunk involvement [28]. Within one month from the start of weakness, two thirds of patients exhibit the first signs of motor function recovery [47]. When this syndrome first manifests, the patient's symptoms will gradually get better over the course of a few weeks to months, and three years later, they will totally disappear [25, 26], nonetheless, supplemental therapies like oral or intravenous corticosteroids and physical therapy can be helpful during this time with as a multidisciplinary approach [11, 17, 43, 44, 48–51]. To put it another way, it was suggested that while most patients are treated conservatively, supportive pain management techniques such opiates and non-steroidal anti-inflammatory medications are helpful during acute periods. Early oral prednisone administration has been suggested by some researchers as a way to slow the disease's progression and promote an early recovery [14]. Some advise utilizing co-analgesics (amitriptyline, carbamazepine, gabapentin) in place of steroids after severe neuropathic pain to avoid the side effects of steroids [52, 53]. Furthermore, by maintaining muscle strength and range of motion, physical rehabilitation therapy also assisted in managing muscle weakness [52]. Patients who don't respond to conservative treatment have access to surgical alternatives [11]. During the first 12 to 18 months after the onset of symptoms, tendon transfers might be a good alternative to restore motion if no meaningful recovery is made [37]. However, it appears that more research is required in this area, given that the majority of studies have only reported on and looked at one case. In this study, we describe a case with similar symptoms, evaluation, treatments performed, treatment results, and a review of similar studies in this field.

## Case description

**Fig. 1:**
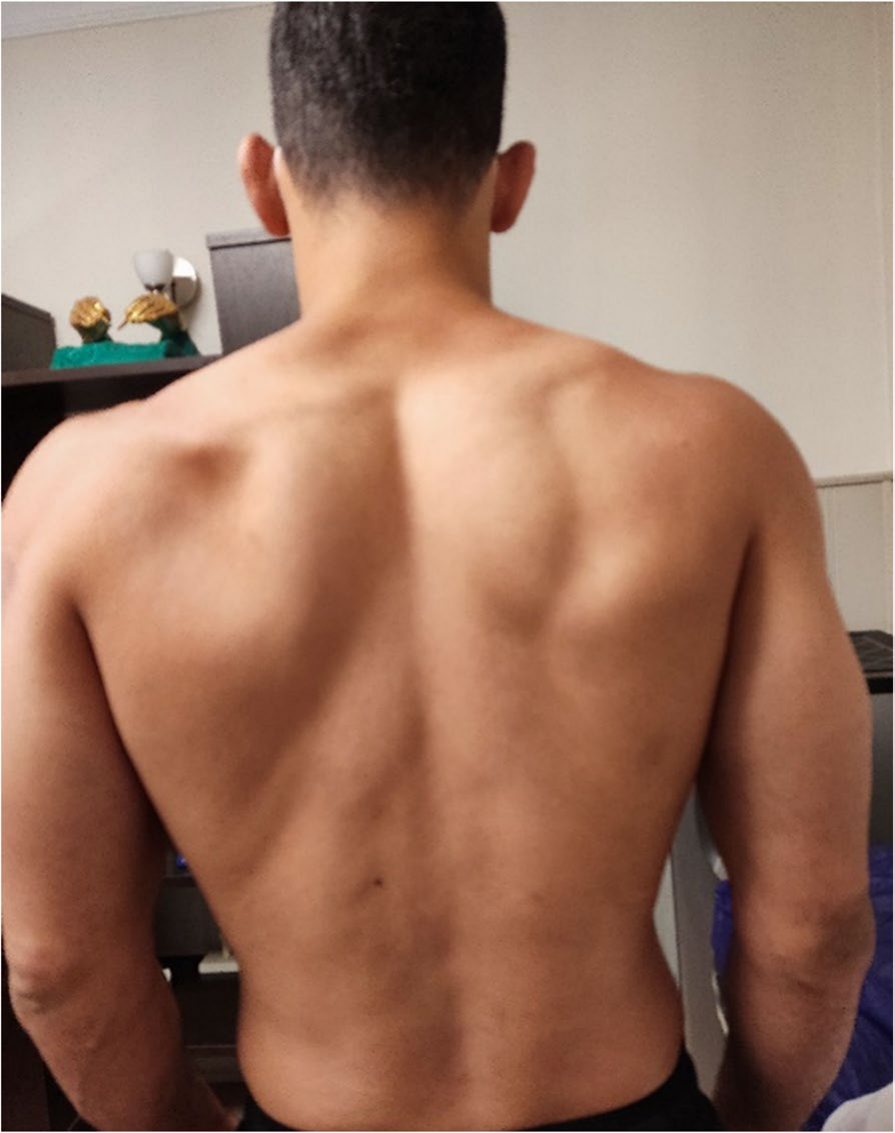
About 3 months after onset

A 24-year-old right-handed professional wrestler with no medical history suddenly developed severe left shoulder pain during sleep two weeks after Sinopharm [Vero Cell]-inactivated COVID-19 vaccine 2 st dose. He experienced severe left shoulder pain and weakness after it for one week, which continued with less intensity after months. After two months, the infraspinatus muscle started to atrophy, and at the time of initial presentation to us, about three months from onset (Fig. 1), all active and passive ROMs were full, and the patient's pain had largely resolved, but muscle strength decreased in abduction and more in external rotation movements. He had no sensory change in his upper extremity. The subject complained of increasing weakness during the competition. His shoulder MRI was normal with no findings of tendinopathy, ganglion cyst, or rotator cuff tear. The first EMG demonstrated a left suprascapular nerve lesion with fair reinnervation in supraspinatus and no reinnervation in infraspinatus (Lt suprasoinatus: ↓ Amplitude, Polyphasic: 2 + , Partial motor unit, and Lt infraspinatus: ↑Insertional activity, Fibrillation: 6/10, Positive Sharp Wave: 6/10, Amplitude: absent, no motor unit.).

The question raised here was: if the suprascapular nerve is under pressure in the scapular notch, why is the infraspinatus muscle atrophied more than the supraspinatus? In addition, there was no evidence of nerve compression in the suprascapular notch or spinoglenoid notch in the patient's MRI. Therefore, for the second time, the subject was referred to a physical medicine and rehabilitation specialist for a more accurate EMG. The findings in the new EMG were: in left supraspinatus muscle: ↑Insertional activity, Fibrillation and Positive Sharp Wave: 2 + and partial voluntary activity; in left infraspinatus muscle: ↑Insertional activity, Fibrillation 2 + , Positive Sharp Wave: 3 + and partial voluntary activity. According to the opinion of the relevant specialist, the subject's history, and his clinical findings, all findings were compatible with the left PTS that affected the upper trunk, mostly the suprascapular nerve, especially the infraspinatus muscle. In order to ensure the desired diagnosis, the subject was referred to a peripheral neurologist for consultation, who also confirmed the desired diagnosis and the planned treatment.

**Fig. 2:**
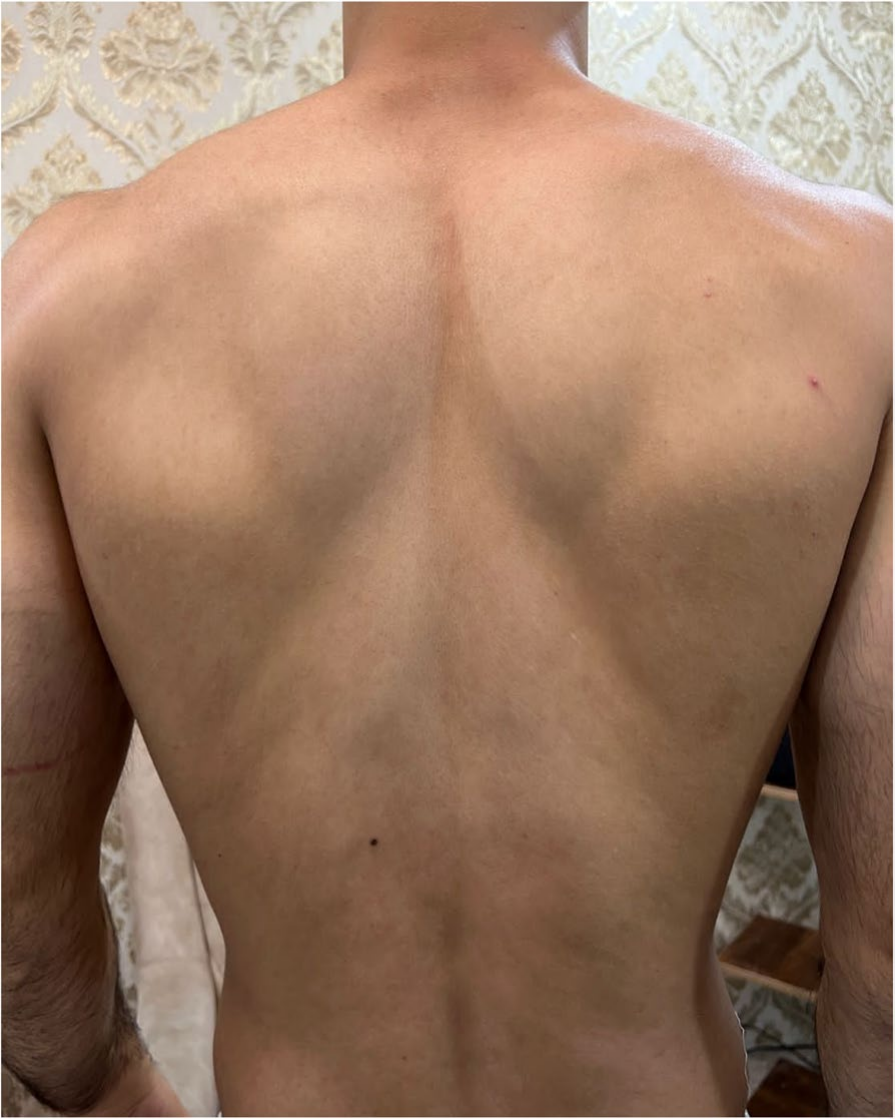
About 1 year after onset

So he was referred to a physiotherapist to maintain ROM and increase muscle strength through electrical stimulation and therapeutic exercises to prevent further muscle atrophy until full nerve regeneration. Exercises to strengthen the muscles include strengthening the supraspinatus and infraspinatus, first actively without weight in the direction of abduction and external rotation three times a day and each time 20 exercises for up to four weeks, then based on the patient's tolerance, using Traband with different elasticity up to three months. Then weight training started with 50% of 1RM and 5% was added to the weight every week or every two weeks according to the patient's ability and continued until the fifth month. In addition, aquatic therapy was suggested to the patient twice a week. After six months, the patient started specialized exercises related to his field under the supervision of the Specialized physiotherapist and the relevant trainer. During this time, he was under our supervision by phone because he lived in another city. According to the patient's statements, five months after the onset of symptoms, muscle strength began to return and the atrophied muscles bulk were gradually filled, and he was able to start specialized wrestling training well and participate in Asian competitions in April 2023, about 11 months after onset. Currently, the subject does his daily activities and professional sports and does not complain of pain or muscle weakness. In the two pictures below, the difference in the atrophic area is evident in about three months (Fig. 1) and almost one year (Fig. 2) after the onset of symptoms.

## Literature review and discussion

NA frequently corresponds to a recent viral upper respiratory tract illness [11, 20]. It is thought that infection causes an abnormal rise in the quantity of antibodies directed against peripheral nerve myelin, which in turn causes inflammation [29]. There is substantial evidence of post-Covid-19 respiratory complications [20]. An increasing number of case reports identified neurological manifestations [54, 55], central [56] and peripheral [57, 58], traumatic [59] and atraumatic [39, 60], as prodromal signs and Covid-19 side effects[61]. According to one theory, the COVID-19 virus may directly infiltrate cells to cause neuropathogens in cases of atraumatic NA. Direct cytotoxic effects on nerves or molecular mimicry are two other potential pathways [62]. There have already been reports of PTS in certain individuals who received the COVID-19 vaccination [17, 42, 44, 48, 50, 63]. As of right now, there's no test that can definitively confirm or rule out PTS on its own. Other differential diagnoses can be ruled out with the use of imaging modalities (MRI, ultrasound) and electrodiagnostic investigation. [52]. We have presented some of related studies in the following titles.

### PTS and COVID-19 infection

#### PTS following COVID-19 vaccination

According to the Table 1, with vaccination and the corona virus, PTS became more common. As a result, while treating both acute and severe shoulder pain, it appears to be regarded as a crucial differential diagnosis. The therapist can make an accurate diagnosis in this case with the aid of a clinical examination, MRI, and EMG [52]. Given the lack of positive findings for tendinopathy, other soft tissue damage, entrapment or nerve damage, and other relevant instances in our case's MRI and EMG results, PTS is the most plausible diagnosis, which may have resulted from an inflammatory response to the vaccine injection. [17]. Though the small number of patients has hampered these investigations, it appears that more research is needed to understand the pathophysiology of this disease.

## Conclusion

According to the research listed in the Tables 1 and 2, it appears that using corticosteroids during the acute phase, is beneficial in reducing the pain and inflammation brought on by lesions after thorough inspection and the rollout of more lesions with comparable symptoms It is also necessary for the patient to be under the supervision of a physiotherapist until complete recovery and to maintain muscles and daily function with the help of therapeutic exercise and electrical stimulation until complete nerve regeneration. What happened to our subject was pleasant, and he returned to his daily and professional life with a full recovery.

**Table 1:** PTS and COVID-19 infection, literature review

Author	Method	Condition	Findings	Intervention	Conclusion
1-Alvarez et al 2021 [20]	Case report	Age: 46 Sex: F Lesion: Lt UL weakness and pain (Proximal > Distal)	NCS/ EMG: - Lt median neuropathy at the wrist - Chronic Lt upper trunk plexopathy with reinnervation MRI: - Brachial plexopathy Hoffman reflex + Diagnosis: - NA syndrome with an atraumatic mechanism after COVID-19	Meloxicam	**↓** Symptoms subsided after 3 months *Upon recheck, she showed normal strength and Hoffman sign
2-O'Sullivan et al. 2021 [64]	A Retrospective Case Series of 15 Patients in Critical Care	Lesion: - Subjects with COVID-19 pneumonia - Had inpatient prone position - Upper limb peripheral nerve injury identified in an acute COVID-19 rehabilitation setting	NCS/ EMG (refer to the main article) MRI X-Ray Diagnosis: - Traumatic Peripheral nerve injuries	-	Prone positioning: ↑ Peripheral nerve injuries Other mechanisms: With neuroinflammatory nature **Recommendation:** Optimizing the prone positioning during inpatient phase
3-Coll et al 2021 [65]	Case report	Age: 63 and 74 Sex: M Lesion: - Rt Shoulder weakness - Amyotrophy related to SAN palsy after COVID-19 infection	NCS: - ↓↓↓CMAP amplitude of upper and lower trapezius muscles EMG: - SAN axonal involvement/ MRI: - Amyotrophy and fatty infiltration X-Ray Diagnosis: - Neuralgic amyotrophy	-	COVID-19 infection: ↑ Trigger for NA as for Guillain–Barré syndrome **Recommendation:** It may seem like being in intensive care is enough of a trauma to cause NA, which needs to be managed
4-Mitry et al 2021 [39]	Case report	Age: 17 Sex: F Lesion: - Joint pain, most prominent in the Lt shoulder and hand - *History of upper respiratory infection	MRI: - ↑T2 signal (supraspinatus, infraspinatus, TMin, TMaj, and trapezius muscles) Bone marrow biopsy: - NL Para-clinic: - ↑ ESR, CRP Diagnosis: - PTS	Oral Steroids	Initial improvement: By oral corticosteroids *SARS-CoV2 (COVID-19) is a post-infectious condition that is becoming a more major potential cause of pain in patients with suspected PTS. The number of cases of COVID-19 cases is increasing globally
5-Díaz et al 2021 [38]	Case report	Age: 36 Sex: M Lesion: - Rt shoulder pain and weakness, for 10 week - *History of upper respiratory infection	Physical examination: - Muscular atrophy (supraspinatus, infraspinatus, deltoids, and biceps) MRI: - ↑T2 signal (supraspinatus and infraspinatus, General atrophy of the periscapular muscles) EMG: - Sub acute and severe degree of Rt brachial plexopathy (upper trunk), - Active denervation and initial reinnervation MRI neurography: - ↑T2 signal in rt Diagnosis: - PTS	Pregabalin (75 mg bid- up to 4 months) Physical Therapy	↑Active joint ranges: After 2 months ↑↑Active ROM and muscle strength in all affected muscle groups: At 4 months, after 8 PT session After 4 months: had home PT At 6 months: No symptoms
6-Zazzara et al 2022 [66]	Case report	Age: 47 Sex: F Lesion: - Unilateral chest pain radiating to the Lt arm for > two months - After Sars-CoV-2 infection	Autoimmune Markers: - NL ECG: - Revealing a sinus rhythm, Pneumological examination: - NL lung function: Muscle strength: - NL Neurological examination: - NL EMG: - NL NCS: - ↓ Lt medial and lateral antebrachial cutaneous nerves Sensory action potential amplitude MRI: - ↑T2 & thickening of the Lt upper trunk Diagnosis: - PTS	Prednisone (12.5 mg, Per day up to two months) Duloxetine & Gabapentin	For many researchers and clinicians, COVID-19 and its neurological ramifications continue to be a learning topic that requires thorough multidisciplinary follow-up **Recommendation:** For these patients to receive the best care possible at a post-acute day hospital, it is essential to understand their requirements, subtleties, and expectations for their post-recovery from Sars-CoV-2 infection
7-Voss et al 2022 [37]	Case report	Age: 61 Sex: M Lesion: - Lt shoulder pain and weakness - History of COVID-19 infection - History of pulmonary embolism - History of Hypertension, and prior arrhythmias - Rotator cuff repair: 2.5 years previously	MRI: - NL EMG: - “Lt brachial plexopathy primarily affecting the upper trunk with evidence of ongoing denervation” X-Ray: - GHJ OA Diagnosis: - PTS	Pain medications (Oral and IV) PT Home-based exercise	Mild pain (at rest and with activity) Weakness: ↓Very gradually up to 8 months from onset After 1 year: ↑↑Function, with persistent overhead weakness **Recommendation:** * Long-term recovery is possible, and the best course of action is usually to wait it out while receiving concurrent physical therapy to keep moving * During the first 12 to 18 months after the onset of symptoms, tendon transfers might be a good alternative to restore motion if no meaningful recovery is made
8-Fortanier et al 2021 [67]	2 Case report	Case 1: Age: 45 Sex: F Lesion: - Rt acute pain and shoulder abduction and elbow flexion weakness - History of SARS-CoV-2 infection several days before shoulder pain Case 2: Age: 21 Sex: M Lesion: - Rt shoulder pain and limitation of elevation - Ten days following SARS-CoV-2 infection, she demonstrate an isolated deficiency of the SM muscle with RT scapula winging	EMG1: - ↓Motor unit recruitment in the biceps brachii MRI1: - ↑Signal involving the Rt C6 root and the superior trunk of the brachial plexus Laboratory analysis1: - NL Diagnosis: - PTS EMG2: - Discrete involvement of the LTN in the SM muscle, exhibiting a neurogenic recruitment pattern MRI2: - ↑Signal involving the Rt LTN Rt shoulder CT-scan: - NL Diagnosis: - PTS	Case1: - No treatment Case2: - Prednisone (Oral at 1 mg/kg, 7 days)	Case1: Three months later: No symptoms Case2: Four months later: Rt shoulder persistence pain, Partial deficit of the serratus major, ↓winging of scapula **Recommendation:** Larger case–control studies are now necessary, for better understanding of the biochemical process causing PTS
9-Ansari et al 2022 [36]	Case report	Age: 54 Sex: M Lesion: - Lt shoulder severe pain and proximal UE weakness - History of moderate-to-severe COVID-19 several days before shoulder pain - History of transplantation of kidneys - Immunosuppressive drugs usage	Clinical examination: - Deep tendon reflexes: Absent in the Lt biceps - lateral arm and forearm: Sensory deficit MRI: - C3–C4 disc bulging, NL plexus EMG: - ↓Sensory radial and median nerves Upper trunk - Brachial plexopathy Laboratory analysis: - NL Diagnosis: - Brachial amyotrophy or PTS	Prednisolone (Oral)	21 days later: Partial strength improvement 2-month later: NL clinical examination with no pain or functional limitations **Recommendation:** Therefore, as additional morbidity brought on by this virus, it is crucial to take into account a potential link between PTS and COVID 19
10-Salomon et al 2022 [68]	Case report	Age: 35 Sex: M Lesion: - Rt shoulder gradually limitation of overhead motions and a "strange" change in Rt scapul - History of neck pain (NPRS 3/10); around Rt upper trapezius area - A broader feeling of numbness during the night emerged after repeatedly tasks	Clinical examination: - ↓↓↓Serratus anterior strength EMG: - Rt LTN: Severe axonal injury - Suprascapular nerve: Minimal injury X-Ray: - NL MRI: - LHBT: Effusion - Sub coracoid bursa - Small cystic lesion in the posterolateral humeral head side Cervical MRI: - NL Diagnosis: A form of neuralgic scapular amyotrophy, or PTS	NSAIDs Rest Conservative rehabilitation with the Physical Therapy (For 6 months)	Six months later: Significant improvement The EMG follow up: Slight reduction of recruitment to the Rt serratus anterior with progressive signs of reinnervation *Physical therapy: Is critical for an effective examination and therapy of a subject complaining of musculoskeletal illnesses mirroring other neurological conditions *Excellent clinical reasoning abilities are necessary for some medical conditions like PTS, which can potentially significantly alter the prognosis for patients and lower the likelihood of a misdiagnosis

*F* female, *M* male, *Lt* Left, *RT* Right, *UL* Upper Limb, *NCS* Nerve Conduction Study, *EMG* Electromyography, *NA* Neuralgic amyotrophy, *MRI* Magnetic Resonance Imaging, *CMAP* Compound Muscle Action Potential, *SAN* Spinal Accessory Nerve, *PTS* Parsonage-Turner, *SyndromeTmin* Teres Minor, *Tmaj* Teres Major, *CRP* C-Reactive Protein, *Bid* Two times a day, *ROM* Range Of Motion, *PT* Physical Therapy, *ECG* Electrocardiogram, *GHJ OA* Glenohumeral Joint Osteoarthritis, *IV* Intravenous, *SM* Serratus Major, *LTN* Long Thoracic Nerve, *CT-Scan* Computerized Tomography, *LHBT* Long Head Biceps Tendon, *NPRS* Numerical Rating Scale

**Table 2:** PTS following COVID-19 vaccination, literature review

Author	Method	Condition	Findings	Intervention	Conclusion
1-Vitturi et al 2021 [44]	Case report and review of the literature	Age: 51 Sex: M Lesion: - Lt UE increasing pain around region of vaccination (first dose of the ChAdOx1-S recombinant vaccine- Vaxzevria, AstraZeneca, Oxford, UK) - 1 m after vaccination: hypoesthesia, abduction and elevation limitation	Clinical examination: - UE proximal muscles atrophy - Deltoid, BB, TB, and infraspinatus muscles paresis EMG (3 months after the onset): - Brachial plexus neuritis - Peripheral neurological damage: Mild to moderate - Some reinnervation: in deltoid, BB, TB, infraspinatus, EPL & EPB, and first interosseous muscles - Lt axillary nerve action potential: ↓Amplitude Diagnosis: PTS	Self-medicate: Paracetamol, NSAIS and Pregabalin Care unit medicate: NSAID, Pregabalin, and PT	Five months later: Partial recovery, slight local muscle weakness *PTS could be an uncommon side effect of the COVID-19 vaccination *This case study demonstrates how crucial it is to be more aware of this link in order to identify and diagnose patients early on and improve therapeutic outcomes
2-Amjad et al 2021 [63]	Case Report and Literature Review	Age: 78 Sex: M Lesion: - Bilateral hand weakness (more in Rt) - History of Pfizer/BioNTech (BNT162b2) COVID-19 vaccine injection, second dose (three weeks before weakness) - History of coronary artery disease	Clinical examination: - ↓Rt-hand grip and wrist flexion strength Laboratory analysis: - NL Brain, cervical spine, and thoracic spine MRI: - NL NCS: - Lt: Brachial plexopathy (lower trunk) - Bilateral median neuropathies at the wrist, - Bilateral ulnar sensory neuropathy EMG: - Bilateral first dorsal interosseous, Rt deltoid, biceps, and triceps muscles: ↓Motor unit recruitment	Prednisone (oral, 40 mg/day) Occupational therapy	↓↓↓Pain Weakness: Slight recovery **Recommendation:** Physicians should be able to take quick action when the number of PTS patients increases with the implementation of a thorough COVID-19 vaccination campaign. With a good prognosis, this usually goes away on its own
3-Chua et al 2022 [17]	Case Report and Literature Review	Age: 64 Sex: M Lesion: - Lt shoulder girdle pain and weakness - History of second COVID-19 vaccine (mRNA-1273; ModernaTX, Inc.; Cambridge, Massachusetts) dose injection )Before the onset of pain( - Be worse 2 weeks later - Lt fourth and fifth digits: Sensory loss with paresthesia and hypoesthesia, as well as on the ulnar aspect of the forearm - History of hypertension and hyperlipidemia	Clinical examination: - Lt finger extensors: ↓Strength in the - Sensation: “Was impaired to light touch distally in the Lt fourth and fifth digits and to a lesser extent in the third digit” EMG: - Lt ulnar SNAP, Lt ulnar-AbdDM CMAPs: ↓Amplitude - Lt EI and flexor digitorum profundus to digit IV: ↓Recruitment pattern, ↑ Lt FDI Spontaneous activity MRI: - ↑Short-TI inversion recovery (STIR) signal - ↑ T2-weighted signal, with mild T1 post contrast enhancement of the medial Lt scalene muscles along the inferior brachial plexus (inflammatory changes and intramuscular edema) Diagnosis: - “Mild, patchy, and acute-to-subacute lower trunk brachial plexopathy”	Prednisone (80 mg/day, Up to 3 days, Followed by a rapid taper of 20 mg decrease per day to off)	One month later: ↑Finger sensation and strength Four months later: Near complete improvement **Recommendation:** To have a better knowledge of PTS's pathophysiology, more research on the condition following COVID vaccination is necessary This is critical because following a COVID-19 immunization, temporary shoulder pain is not uncommon. If a subject experiences shoulder pain in addition to weakening or changes in sensation in their affected extremity, PTS should be considered a possibility
4-Shields et al 2022 [42]	Case Series Clinical and EMG Findings in 6 Patients	Lesion: - Shoulder pain and weakness (5 ipsilateral side to the injection site and 1 contralateral side 2 after 1st dose of the vaccine and 4 after the 2nd vaccine dose) Vaccination history: - 4 subjects: Pfizer-BioNTech COVID-19 vaccine - 2 subjects: Moderna COVID-19 vaccine prior to symptom onset (Mean duration: 17 days, range: 5 days–8 weeks)	EMG: - 3 subjects: Upper trunk/lower trunk involvement - 1 subjects: Posterior cord - 1 subjects: AIN - 1 patient: PIN Cervical MRI in 5 subjects: - NL - Subject #1, brachial plexus MRI: No abnormalities of the brachial plexus Diagnosis: PTS	Prednisone/prednisolone in, gabapentin 4 subjects: PT	Pain in all 6 subjects: Improvement or near complete treatment Arm/hand muscle strength: 3 subjects no improvement, 3 subjects with some recovery **Recommendation:** Although total healing may not always happen, the best results are provided by early detection of this ailment and treatment with corticosteroids and PT

*F* female, *M* male, *Lt* Left, *RT* Right, *UL* Upper Limb, *NCS* Nerve Conduction Study, *EMG* Electromyography, *NA* Neuralgic amyotrophy, *BB* Biceps Brachii, *TB* Triceps Brachii, *EPL* Extensor Pollicis Longus, *NSAID* Non-Steroidal Anti-Inflamatory Drugs, *SNAP* Sensory Nerve Action Potential, *AbdDM* Abductor Digiti Minimi, *EI* Extensor Indices, *FDI* First Dorsal Interosseus, *AIN* Anterior Interosseous Nerve, *PIN* Posterior Interosseous Nerve

## Data Availability

All data generated or analysed during this study are included in this published article.

## Abbreviations

BN: Brachial plexus Neuritis
PTS: Parsonage-Turner syndrome
NA: Neuralgic Amyotrophy
